# Polysaccharides from the Chinese medicinal herb *Achyranthes bidentata *enhance anti-malarial immunity during *Plasmodium yoelii *17XL infection in mice

**DOI:** 10.1186/1475-2875-11-49

**Published:** 2012-02-20

**Authors:** Xiaotong Zhu, Yanyan Pan, Li Zheng, Liwang Cui, Yaming Cao

**Affiliations:** 1 Department of Immunology, College of Basic Medical Sciences, China Medical University, No.92, Bei'er Road, Heping District, Shenyang, Liaoning 110001, China; 2 Institute of Pathology and Pathophysiology, China Medical University, No.92, Bei'er Road, Heping District, Shenyang, Liaoning 110001, China; 3 Department of Entomology, Pennsylvania State University, 501 ASI Building, University Park, Pennsylvania 16802, USA

**Keywords:** *Achyranthes bidentata *polysaccharides, *Plasmodium yoelii *17XL, Immune responses, Immuno modulatory effect

## Abstract

**Background:**

Clinical immunity to malaria in human populations is developed after repeated exposure to malaria. Regulation and balance of host immune responses may lead to optimal immunity against malaria parasite infection. Polysaccharides (ABPS) derived from the Chinese herb ox knee *Achyranthes bidentata *possess immuno-modulatory functions. The aim of this study is to use the rodent malaria model *Plasmodium yoelii *17XL (*P. y*17XL) to examine whether pretreatment with ABPS will modulate host immunity against malaria infection and improve the outcome of the disease.

**Methods:**

To determine whether ABPS could modulate immunity against malaria, mice were pretreated with ABPS prior to blood-stage infection by *P. y*17XL. Host survival and parasitaemia were monitored daily. The effect of pretreatment on host immune responses was studied through the quantitation of cytokines, dendritic cell populations, and natural regulatory T cells (Treg).

**Results:**

Pretreatment with ABPS prior to infection significantly extended the survival time of mice after *P. y*17XL infection. At three and five days post-infection, ABPS pretreated mice developed stronger Th1 immune responses against malaria infection with the number of F4/80^+^CD36^+ ^macrophages and levels of IFN-γ, TNF-α and nitric oxide being significantly higher than in the control group. More importantly, ABPS-treated mice developed more myeloid (CD11c^+^CD11b^+^) and plasmacytoid dendritic cells (CD11c^+^CD45R^+^/B220^+^) than control mice. ABPS pretreatment also resulted in modulated expression of MHC-II, CD86, and especially Toll-like receptor 9 by CD11c^+ ^dendritic cells. In comparison, pretreatment with ABPS did not alter the number of natural Treg or the production of the anti-inflammatory cytokine IL-10.

**Conclusion:**

Pretreatment with the immuno-modulatory ABPS selectively enhanced Th1 immune responses to control the proliferation of malaria parasites, and prolonged the survival of mice during subsequent malaria infection.

## Background

Malaria is a serious infectious disease caused by protozoan parasites of the genus *Plasmodium*. Worldwide, there are estimated 200-300 million malaria cases annually, causing approximately one million deaths, mainly in children [1]. The fatalities are normally associated with severe malaria involving life-threatening complications such as acute respiratory distress, metabolic acidosis and cerebral malaria. Despite treatment with the best available anti-malarial drugs and supportive care, severe malaria is fatal in 10-30% of patients [2,3]. Additional adjunctive management strategies are being considered to reduce the severity of disease.

In endemic areas, repeated exposure to malaria infection results in the development of clinical immunity to disease, which is characterized by low parasitaemia levels rather than sterilizing immunity. Therefore, the primary focus of malaria vaccines is to reduce morbidity and mortality associated with this disease. Better understanding of host immunity against malaria is needed to achieve this goal. Immunity to asexual blood stages of malaria parasites involves both cellular and antibody-mediated mechanisms [4]. Although cell-mediated immune responses play the major role in protection against parasite replication, they also result in detrimental inflammation and contribute to induction of severe disease. CD4^+ ^T cells govern the cell-mediated immune response to malaria parasites through the production of cytokines. Pro-inflammatory (Th1) and anti-inflammatory (Th2) cytokines produced by CD4^+ ^T cells play decisive roles in the outcome of malaria infections. Early pro-inflammatory cytokine responses seem to mediate protective immunity, whereas late in infection they appear to contribute to pathology [5]. In mice, the anti-inflammatory cytokine IL-10, produced by natural regulatory T cells (Treg), is required to limit pro-inflammatory immune responses [5-7]. Meanwhile, as the pacemaker of the immune response, dendritic cells (DCs) provide a source of cytokines that contribute to shaping up cell-mediated and humoral immunity by inducing Th1/Th2 differentiation of T cells and antibody production by B cells [8,9]. In addition to DCs, another important effector molecule in malaria is the toxic free radical NO, which is widely recognized as an important biological mediator [10]. In the context of an acute inflammatory disease, such as malaria, much of the NO produced comes from the cytokine inducible NO synthase (iNOS) present in monocytes, macrophages, and neutrophils [11]. Pro-inflammatory cytokines (e.g. IFN-γ and TNF-α) increase iNOS-generated NO production, whereas anti-inflammatory cytokines (e.g. IL-10 and TGF-β) down-regulate NOS2 expression [11]. NO also has been reported to have potent parasiticidal properties against *P. falciparum *[12] and can thereby limit parasitaemia [13]. These findings highlight the significance of a crucial balance in the inflammatory response for controlling infection and preventing pathology in malaria. Thus, regulation of host immune responses may lead to enhanced immunity against malaria parasite infection.

Polysaccharides are high molecular weight compounds formed by repeating sub-units of sugars, which are widely distributed in plants, animals and microbes [14-16]. Polysaccharides have been shown to possess anti-tumour, anti-hepatitis and anti-senile effects, as well as immunomodulatory functions, such as stimulating splenocyte proliferation while in combination with LPS, activating macrophage, NK T cells, B cells and maturation of DCs [17,18]. Ox knee (*Achyranthes bidentata*) is a traditional Chinese medicinal herb, which has anti-inflammatory activities and is used to "nourish the kidney and liver, drain dampness and promote circulation". The purified *A. bidentata *polysaccharides (ABPS) are water-soluble molecules with its unit composition molecular weight of ~1400 D, and contain fructose and glucose residues in the molar ratio of 8:1. ABPS are bioactive and have many important functions such as anti-tumour, anti-inflammation and anti-aging [19-22]. The immuno-modulatory effect of ABPS has prompted us to investigate whether pretreatment with ABPS affects the host immunity against malaria infection. Here, the murine malaria model was used with the lethal strain of *Plasmodium yoelii *17XL (*P. y*17XL). In this model, mice are unable to establish an effective Th1 response and die during the early stages of infection [23,24]. The results indicated that pretreatment with ABPS could boost host Th1 response against the parasite and prolong the survival of infected mice.

## Methods

### Mice, parasites and experimental infection

Female BALB/c mice, aged six to eight weeks, were purchased from Beijing Animal Institute (Beijing, China). *P. y*17XL parasitized erythrocytes were purified as previously described [25]. To initiate malaria infection, each mouse was injected intraperitoneally (i.p.) with 1 × 10^6 ^*P. y*17XL parasitized erythrocytes per mouse. Parasitaemia was monitored by light microscopy. Mortality of infected mice was monitored daily. All experiments were performed in compliance with the local Institutional Animal Care and Use Committee.

### ABPS treatment

White powder ABPS with 99% purity was kindly provided by Gengyuan Tian of Shanghai Institute of Organic Chemistry, Chinese Academy of Sciences, Shanghai, China. ABPS injection solutions were prepared with physiological saline. To study the effect of ABPS on parasitaemia and host survival, 0.1 ml ABPs solutions were injected i.p. daily into mice at the dosage of 50 mg/kg for consecutive 10 and 15 days before or immediately after *P.y*17XL infection. This dosage was adopted based on an earlier report, which shows that 50 mg/kg of ABPS is optimal against Lewis lung cancer in C57BL/6 mice [22]. The control group received the same volume of physiological saline at the same time points. Subsequently, to study the immuno-modulatory effect of ABPS, two dosages of ABPS (10 and 50 mg/kg) were used for i.p. injection daily into the mice for consecutive 10 days before infection [22,26].

### Splenocyte preparation, culture and measurement of cytokines and nitric oxide

Splenocyte culture was performed as previously described [27]. Briefly, spleens from uninfected and infected mice were removed aseptically and pressed through a sterile fine-wire mesh with 10 ml RPMI 1640 supplemented with 10% heat-inactivated foetal calf serum (FCS), 25 mM Hepes, 0.12% gentamicin and 2 mM glutamine. Cell suspensions were collected by centrifuging at 350 *g *for 10 minutes. Cell viability was determined by Trypan Blue exclusion and was > 90%. Aliquots of the cell suspensions containing 5 × 10^6 ^cells in 500 μl were incubated in 24-well flat-bottom tissue culture plates in triplicate for 48 hours at 37°C in a humidified 5% CO_2 _incubator. Supernatant fractions were collected and stored at -80°C. Levels of IFN-γ, TNF-α and IL-10 were measured by commercial enzyme-linked immuno-sorbent assay (ELISA) kits according to the manufacturer's protocol (R&D Systems). As a measure of nitric oxide (NO) production, concentrations of NO_2_^- ^in cell supernatants were determined by the Griess reaction [27].

### Flow cytometry analysis

All antibodies used for flow cytometry were purchased from BD Biosciences or eBioscience (San Diego, CA, USA) unless otherwise indicated. To assess natural Treg, spleen cells were double-stained with FITC-conjugated anti-CD4 monoclonal antibody (mAb) and PE-conjugated anti-CD25 mAb. After fixation and permeabilization, cells were stained with APC-conjugated anti-Foxp3 mAb. To distinguish the sub-sets of DCs, spleen cells were double-stained with FITC-conjugated anti-CD11c mAb and PE-conjugated anti-CD11b mAb or PerCP-conjugated anti-B220 mAb. To measure the maturation markers of DCs, spleen cells were double-stained with FITC-conjugated CD11c mAb and PE-conjugated anti-CD80, CD86, CD40 or MHC-II mAb. To determine the expression of Toll-like receptor 9 (TLR9) within DCs, spleen cells were first stained with FITC-conjugated CD11c mAb, fixed, permeabilized and probed with biotinylated anti-TLR9 mAb followed by PE-conjugated streptavidin. Macrophages were analysed by staining spleen cells with FITC-conjugated F4/80 mAb and/or PE-conjugated anti-CD36 mAb. After staining, the cells were washed twice with PBS containing 1% foetal calf serum and suspended in 500 μl of PBS. All cells were analysed by flow cytometry using a FACSCalibur™ flow cytometer with the CellQuest software version 3.3 (BD Biosciences, Franklin Lakes, NJ, USA). Viable cells were gated by forward and side scattering [28,29].

### Statistical analysis

Statistical significance of the results was analysed by one-way ANOVA. Time-to-event data were statistically analysed with the Kaplan-Meier approach to survival analysis (SPSS 17.0). A value of *P *< 0.05 was considered significant.

## Results

### ABPS extends survival of *Plasmodium yoelii *17XL infected mice

**Figure 1:**
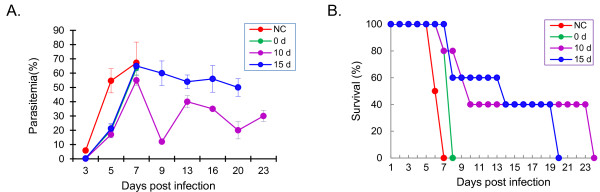
**Effects of pretreatment with ABPS on parasitaemia (A) and survival rate (B) of infected mice**. ABPS was injected i.p. into mice at the dosage of 50 mg/kg for consecutive 10 (10 d) or 15 days (15 d) before and immediately after (0 d) *P.y*17XL infection. The control group (NC) received the same volume of physiological saline at the same time points. Data presented as the mean with standard error (n = 10 mice per group)

To determine the effects of ABPS on *P.y*17XL infection in BALB/c mice, parasitaemia and survival rate were monitored daily after infection. In the control group, parasitaemia rose sharply to >50% on day 5 and ~60% on day 7, and all mice died of high parasitaemia at five to seven days post-infection (dpi) (Figure 1), consistent with our previous reports [24,30]. In all ABPS-treated groups, parasitaemia on day 5 was significantly lower than that of the control group (*P *< 0.05). In mice treated with ABPS for 10 and 15 days, parasitaemia also peaked on day 7, but declined subsequently (Figure 1A). Whereas all mice treated with ABPS at the time of infection died from high parasitaemia at 8 dpi, pretreatment with ABPS prior to malaria infection significantly extended the survival time of infected mice (*P *< 0.01, Kaplan-Meier's statistics). Specifically, 40% of mice still survived at 19 and 23 dpi in groups pretreated with ABPS for 10 and 15 days, respectively (Figure 1B). This result showed that pretreatment with ABPS before infection with *P.y*17XL could suppress the progression of malaria in BALB/c mice. Based on this result, all subsequent immunology experiments used the treatment time of 10 days before infection and dosages of 10 and 50 mg/kg of ABPS.

### ABPS have immuno-regulatory effects on adaptive immune responses

**Figure 2:**
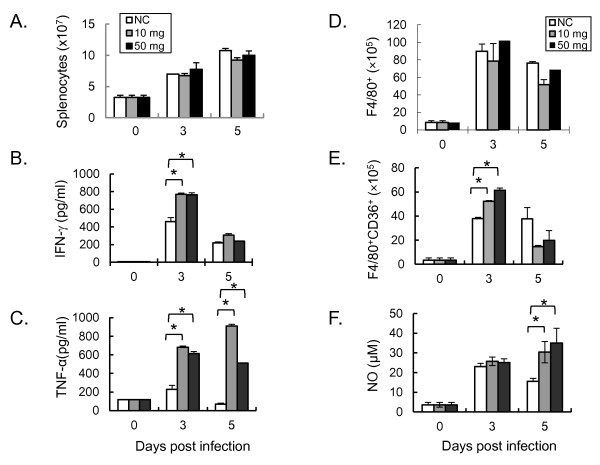
**The effects of ABPS pretreatment on immune responses during early *Plasmodium yoelii 17XL (P. y*17XL) infection**. Mice were injected i.p. with two different dosages of ABPS (10 mg/kg and 50 mg/kg) for consecutive 10 days prior to infection, and spleen lysates were prepared on days 0, 3 and 5 after infection. The control group (NC) received the same volume of physiological saline at the same time points. **(A) **Total number of splenocytes per mouse. **(B) **Levels of IFN-γ **(C) **Levels of TNF-α **(D) **Total number of F4/80^+ ^macrophages. **(E) **Total number of CD36+ macrophages. **(F) **Concentration of NO_2_-. Values represent the mean with standard error (n = 3 mice per group). Results are representative of three independent experiments. Asterisk (*) indicates statistically significant difference (*P *< 0.05) between the control and ABPS pretreated groups of mice. Open, grey and black boxes indicate control group (NC), 10 mg/kg ABPS group (10 mg), and 50 mg/kg ABPS group (50 mg), respectively

The effects of ABPS pretreatment on adaptive immune responses were investigated during early infection by *P.y*17XL in BALB/c mice. Although ABPS pretreatment did not significantly alter the number of splenocytes (Figure 2A), it caused a remarkable induction of IFN-γ at 3 dpi (Figure 2B), and TNF-α at 3 and 5 dpi as compared to the control group (Figure 2C, *P *< 0.05). Whereas the number of F4/80^+^macrophages did not differ remarkably between the ABPS-treated and control groups (Figure 2D), there were significantly more F4/80^+^CD36^+ ^macrophages in the ABPS-treated groups at 3 dpi (Figure 2E). As a result, in the ABPS-treated groups, NO synthesis was also significantly increased at 5 dpi (*P *< 0.05, Figure 2F).

### ABPS do not affect the response of natural Treg cells during *Plasmodium yoelii *17XL infection

**Figure 3:**
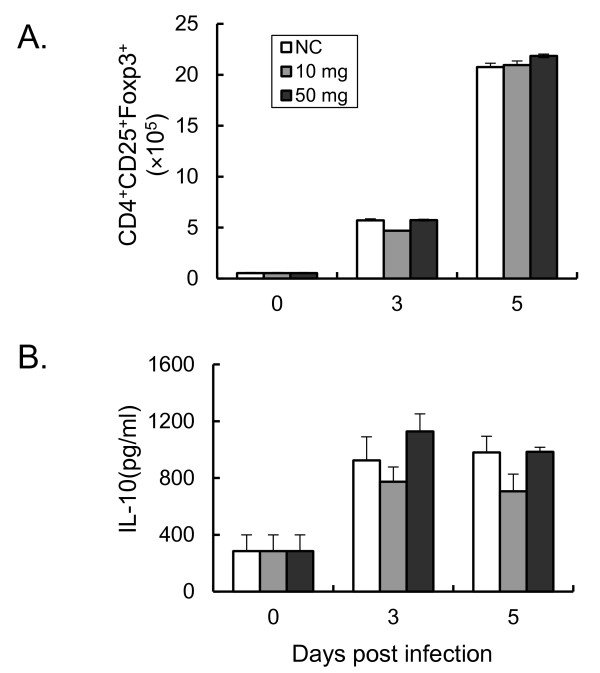
**The effect of ABPS pretreatment on the response of natural Treg**. **(A) **Numbers of Treg (CD4^+^CD25^+^Foxp3^+^) cells in the spleens of infected and control mice quantified by flow cytometry. **(B) **Spleen cell culture suspensions were collected on different days after infection and levels of IL-10 protein were measured by ELISA. Data presentation is the same as in Figure 2

To elucidate how ABPS activated the immune response, the potential regulation of response of natural Treg by ABPS during activation of immune responses was investigated *in vivo*. Previous studies have shown that proliferation of Treg is causally associated with the suppression of Th1 responses during early malaria infection, leading to increased parasitaemia and mortality in mice [30]. In control mice, the absolute number of natural Treg increased from 5 × 10^5 ^at 3 dpi to 2 × 10^6 ^at 5 dpi. This increase of Treg population was not significantly affected by pretreatment with ABPS (*P *> 0.05, Figure 3A). Similarly, pretreatment with ABPS did not affect the production of IL-10 (*P *> 0.05, Figure 3B)

### ABPS increase TLR9 expression in CD11c^+ ^DCs

**Figure 4:**
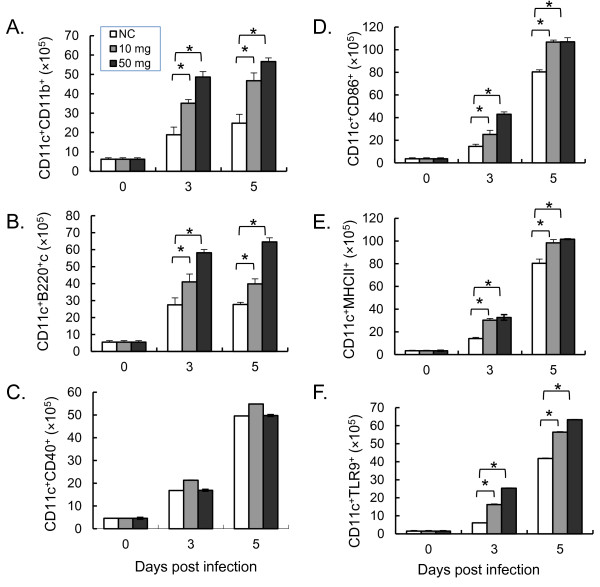
**ABPS induced DC differentiation and maturation and increased TLR9 expression on DCs**. **(A) **The number of mDCs. **(B) **The number of pDCs. **(C) **The number of CD11c^+ ^cells expressing CD40. **(D) **The number of CD11c^+ ^cells expressing CD86. **(E) **The number of CD11c^+ ^cells expressing MHC-II^+^. **(F) **The number of CD11c^+ ^cells expressing TLR9. Data presented as the mean with standard error (n = 3 mice per group). Data presentation is the same as in Figure 2

Since ABPS had no effect on the percentage of natural Treg, the effects of ABPS on DCs, which are essential for the induction of T cell responses, were examined. In the ABPS-treated groups, there were significant increases (*P *< 0.05) in the proportions of myeloid DCs (mDCs) and plasmocytoid DCs (pDCs) (Figure 4A, B). Whereas pretreatment with ABPS did not affect the expression of CD40 or CD80 (Figure 4C), it significantly enhanced the expression of DC maturation markers CD86 (Figure 4D) and MHC II (Figure 4E) on DCs. Strikingly, the results showed that ABPS treatment significantly increased the expression of TLR9 by DCs (*P *< 0.05, Figure 4F).

## Discussion

The Chinese herbal medicine ox knee (*A. bidentata*) possesses pro-inflammatory activities, and polysaccharides from this herb have been shown to enhance immunity [19]. This study demonstrated that pretreatment with ABPS could delay the progression of malaria in mice, probably through boosting of host immunity. It was also found that ABPS could effectively control parasitaemia levels and extend host survival. ABPS treatment enhanced the production of IFN-γ, TNF-α and NO, and increased the number of F4/80^+^CD36^+ ^macrophages. ABPS also promoted the differentiation and activation of DCs with increased expression of maturation markers. This study further verified the immuno-modulatory activity of ABPS during the early stage of *P.y*17XL infection in this murine malaria model, which provided some insights into the regulatory mechanisms by which ABPS mediate anti-malarial protection.

During malaria infection, effective Th1 immune responses at the early stages of infection critically influence the later development and final outcome of the disease [31-34]. IFN-γ is found to be necessary during the resolution of primary infection and for limiting parasite replication in early phase of the infection [35,36]. In current study, it was found that pretreatment with ABPS significantly stimulated IFN-γ, TNF-α and NO production *in vivo *during *P.y*17XL infection, which might be responsible for the observed improvement in the extended survival in ABPS-treated mice.

DCs provide a critical link between the innate and adaptive immune responses. Increased expression of co-stimulatory molecules, which is characteristic of maturation of DCs, is crucial to the activation of T cells [37]. These molecules have been shown to play an important role in murine malaria models. High expression of MHC-II molecules is crucial for DCs to present antigens to CD4^+ ^T cells. It has been found that blocking the CD80/CD86 signaling pathway disrupts the Th1/Th2 balance in the *Plasmodium chabaudi *AS malaria model [38]. It is reported that ABPS can induce murine DCs phenotypic maturation *in vitro *as revealed by increased expression of CD86, CD40 and MHC-II [18]. In this study, the significant up-regulation of co-stimulatory molecules on the surface of CD11c^+ ^cells in ABPS-treated mice was detected, indicating that ABPS treatment could significantly improve the maturation of DCs.

TLRs are expressed on or within innate immune cells including DCs, and recognize pathogen-associated molecular patterns from different microorganisms [39]. Accumulating evidence suggests that TLRs are also involved in both rodent and human malaria [40-42]. It has been reported that TLR9 responds to haemozoin and parasite protein-DNA complex released from the parasitized erythrocytes [41,43,44], and TLR9 polymorphisms are associated with disease manifestation in malaria [37]. The significantly elevated expression of TLR9 in DCs in *P.y*17XL-infected mice pretreated with ABPS suggests that TLR9 might be essential for ABPS-mediated immuno-regulation during malaria infection.

Increasing evidence indicates that innate immune responses contribute to the control of blood-stage malaria infection, reduced parasite burden, and slowing down of the progression to severe disease. Macrophages have a remarkable non-specific ability to phagocytize and kill protozoan parasites. Both human and rodent macrophages effectively phagocytize *P.y*17XL-parasitized erythrocytes [45,46]. Furthermore, the class B family of scavenger receptor CD36 on macrophages is also implied in protection against malaria, since CD36-deficient individuals are at a greater risk of developing severe and cerebral malaria [47]. Whereas the total number of macrophages (F4/80^+^) in ABPS-pretreated mice was relatively unchanged, the number of F4/80^+^CD36^+ ^macrophages significantly increased at 3 dpi in ABPS pretreated mice. As such, the increase in the number of CD36^+ ^macrophages might be involved in phagocytosis of parasitized erythrocytes, leading to reduced parasitaemia at 5 dpi in ABPS-pretreated mice.

Natural Treg expand during *Plasmodium *infection [5,24,48,49] and have been shown to inhibit the development of Th1 immune responses [3,24], leading to persistent rise of parasitaemia [24,50]. Here it was found that ABPS only improved the activation of DCs, which may improve the initiation of an antigen-specific immune response, whereas no effect was observed on Treg or production of the anti-inflammatory cytokine IL-10. This suggests that the immuno-regulatory effects of ABPS may be selective on boosting different host immune responses.

Taken together, the current study further confirmed the immuno-regulatory effects of ABPS. The results have demonstrated that ABPS could activate immune responses against malaria blood-stage infection in mice and that this effect may be mediated by stimulation of DC maturation and activation of F4/80^+^CD36^+ ^macrophages, which may phagocytose parasitized erythrocytes and release pre-inflammatory cytokines. These findings laid the foundation for further testing the potential benefits of administering ABPS as an "immuno-prophylaxis" to inhibit preemptively the progression of malaria infection.

## Conclusions

The aim of this study is to determine whether pretreatment of hosts with immuno-modulatory polysaccharides from the Chinese medicinal herb ox knee, could affect host immunity during malaria infection in a rodent malaria model. It was found that pretreatment of mice with ABPS could boost host Th1 immune responses during early *P.y*17XL infection, which includes elevated numbers of activated macrophages and Th1 cytokines. In addition, ABPS pretreatment promoted the maturation of DCs, which are essential in development of parasite-specific adaptive immunity. Yet, the treatment did not alter the number of Treg and the level of anti-inflammatory cytokine IL-10. Findings from this study underscore the potential of administering immuno-modulatory compounds like ABPS to strengthen host immunity against malaria.

## Competing interests

The authors declare that they have no competing interests.

## Authors' contributions

XZ carried out the flow cytometry, statistical analysis and drafted the manuscript. YP performed splenocytes preparation and culture, detection of cytokines and NO_2_^- ^concentration. YC and LC conceived the study and participated in the design of the study. All authors read and approved the final manuscript.
